# Long-lasting microbial larvicides for controlling insecticide resistant and outdoor transmitting vectors: a cost-effective supplement for malaria interventions

**DOI:** 10.1186/s40249-020-00767-3

**Published:** 2020-11-26

**Authors:** Guofa Zhou, Eugenia Lo, Andrew K. Githeko, Yaw A. Afrane, Guiyun Yan

**Affiliations:** 1 Program in Public Health, University of California, Irvine, CA 92697 USA; 2 Department of Biological Sciences, University of North Carolina, Charlotte, NC 28223 USA; 3 Central for Global Health Research, Kenya Medical Research Institute, Kisumu, Kenya; 4 Department of Medical Microbiology, University of Ghana, Accra, Ghana

**Keywords:** Long-lasting microbial larvicide, Cost-effectiveness, Supplemental tool, Malaria control and elimination

## Abstract

The issues of pyrethroid resistance and outdoor malaria parasite transmission have prompted the WHO to call for the development and adoption of viable alternative vector control methods. Larval source management is one of the core malaria vector interventions recommended by the Ministry of Health in many African countries, but it is rarely implemented due to concerns on its cost-effectiveness. New long-lasting microbial larvicide can be a promising cost-effective supplement to current vector control and elimination methods because microbial larvicide uses killing mechanisms different from pyrethroids and other chemical insecticides. It has been shown to be effective in reducing the overall vector abundance and thus both indoor and outdoor transmission. In our opinion, the long-lasting formulation can potentially reduce the cost of larvicide field application, and should be evaluated for its cost-effectiveness, resistance development, and impact on non-target organisms when integrating with other malaria vector control measures. In this opinion, we highlight that long-lasting microbial larvicide can be a potential cost-effective product that complements current front-line long-lasting insecticidal nets (LLINs) and indoor residual spraying (IRS) programs for malaria control and elimination. Microbial larviciding targets immature mosquitoes, reduces both indoor and outdoor transmission and is not affected by vector resistance to synthetic insecticides. This control method is a shift from the conventional LLINs and IRS programs that mainly target indoor-biting and resting adult mosquitoes.

## Background

There has been a massive scale-up of antimalarial interventions since 2000 including long-lasting insecticidal nets (LLINs), indoor residual spraying (IRS), and artemisinin-based combination therapy (ACT). These interventions have led to significant reductions in malaria morbidity and mortality [1]. However, resurgence in malaria morbidity has been observed in some African countries in the past few years despite a high LLINs coverage [1, 2]. It is apparent that the existing front-line vector control measures fail to break the transmission cycle of malaria parasites in many malaria-endemic areas [3–8]. Persistence and resurgence of the vector mosquito populations continues to be a challenging issue for malaria control and elimination. Resistance to synthetic insecticides, particularly pyrethroids and outdoor parasite transmission have become a major hurdle to malaria control, prompting the World Health Organization (WHO) to call for the development and adoption of viable alternative methods of malaria vector control that can reduce the reliance on synthetic insecticides.

While pyrethroid and other chemical insecticides have been used for two decades for disease vector and/or agricultural pest control in malaria endemic Africa, there is ample evidence of the emergence and spread of pyrethroid resistance in the major African malaria vectors *Anopheles gambiae*, *An*. *arabiensis*, and *An*. *funestus* [9–12]. Unfortunately, pyrethroids are the only class of insecticides that the WHO recommends for the treatment of insecticide-treated nets (ITNs). Outside Africa, chemical insecticide resistance has also been detected in other major malaria vectors such as *An*. *minimus*, *An*. *dirus*, *An*. *sinensis*, and *An*. *maculatus* in Asia, as well as *An*. *darlingi* in South America [12]. Resistance to multiple chemical insecticides in malaria vectors has been observed in different locations [7, 8]. The scale-up of LLINs and IRS programs has unequivocally selected for increased insecticide resistance [12].

Outdoor malaria transmission has become a very important challenge to malaria control [13–15]. The current front-line malaria vector control programs such as LLINs and IRS target only indoor biting and resting mosquitoes. However, a number of recent studies have documented changes in the biting behaviour of *An*. *gambiae* and *An*. *funestus*, from biting exclusively indoors at night to biting both indoors and outdoors during early evening and morning hours when people are not protected by IRS or LLINs, or biting indoors but resting outdoors [7, 8]. These behavioural changes in the mosquitoes have unquestionably challenged the effectiveness of existing control programs that primarily target indoor mosquitoes and urged the need of an expanded or alternative vector control toolkit.

Given that outdoor transmission and insecticide resistance compromises the efficacy of LLINs and IRS [7], additional vector control tools that target outdoor biting and resting mosquitoes are urgently needed to further reduce malaria transmission. Developing alternative interventions with a long-lasting impact will reduce intervention operation cost, and thus enhancing sustainability [16]. Benelli and Beier have recently published a review on the development of further tools for effective mosquito vector control [7]. Killeen et al. further discussed evidence-based development of new vector control strategies from a programmatic point of view [8]. However, many of the proposed tools are still in development or in conceptual stage [7, 8]. Intervention measures targeting adult vectors such as topical or spatial repellents, attractive toxic sugar baits (ATSB) and outdoor mosquito traps are a few examples of tools that have been tested in the field for their effectiveness and applicability [7, 8]. Meta-analysis and field trial results suggest that topical/spatial repellents or outdoor light traps are not very effective in reducing outdoor transmission [17–19]. ATSB methods have been reported as highly effective and target-specific, but more field tests are required to determine its efficacy and cost-effectiveness on malaria incidence reduction [7]. Larval source management, including larviciding targeting immature-stage vectors may reduce overall vector population both indoors and outdoors [20–26]. *Bacillus thuringiensis israelensis* (*Bti*) and *Bacillus sphaericus* (*Bs*) based bacterial agents are considered as highly effective microbial mosquito larvicide, which targets aquatic stages and thus reduces both indoor and outdoor mosquitoes. They can be used either individually or as a mixture, and have been shown to be safe to non-target organisms cohabiting with the mosquito larvae in the natural environment [27]. Further investigations on their efficacy and cost-effectiveness are still underway.

## Main text

### Challenges in the role of larval mosquito control

#### Effectiveness of larviciding

Larval control and environmental management have played prominent roles in malaria elimination in the past [28, 29]. In the US and Europe, larval control especially larviciding has been the preferred vector control tool for many years and is still the primary tool in use today [29]. Larviciding has been shown to be effective in killing mosquito larvae and reducing adult abundance [24, 25].

Currently, United States Environment Protection Agency (US EPA) registered three major types of larval control agents, i.e., microbial larvicides, insect growth inhibitors, and chemical insecticide (mainly temephos). Microbial larvicides, *Bti* and *Bs* inhibit food digestion of the mosquito larvae and thus prevent larval development. Insect growth inhibitors such as methoprene and hydroprene are structurally related to insect juvenile hormone, preventing mosquito larvae from maturing into adults or delays egg maturation [12]. However, methoprene and S-methoprene show some toxicity to some fish and aquatic invertebrates in laboratory tests [30, 31]. Temephos, an organophosphate insecticide, causes rapid neurotoxicity to mosquitoes. However, in areas with long-term applications, mosquitoes have developed high resistance to temephos [32–34]. Microbial larvicides so far are the preferred larvicides over chemical adulticides for mosquito control.

Microbial larviciding has several advantages over chemical adulticides. First, microbial larvicides target mosquito larvae living in confined breeding habitats, so the effectiveness is not influenced by the changing biting and resting behaviours of adult mosquitoes. Second, larval control provides the dual benefit of reducing the number of house-entering mosquitoes as well as the number of mosquitoes that bite and rest outdoors. Third, when compared to pyrethroid or other chemical insecticides, microbial larvicides have different modes of action against mosquitoes. There is no cross-resistance between chemical insecticide and microbial larvicide [35, 36]. In addition, microbial larvicides are currently considered the safest biological insecticides for the health of humans and other non-target organisms. Fourth, larval control does not conflict with but complements the front-line LLINs and IRS malaria control programs, given that those methods target different stages of vector development. Fifth, microbial larviciding provides great collateral benefits because it kills all species of mosquito larvae, including *Anopheles*, *Culex* and *Aedes* and other disease vectors [12].

#### Key limitation of current larvicide formulations

While larval control may be one of the solutions to reduce outdoor as well as overall malaria transmission, microbial larviciding has several limitations as it is practiced today [26, 27]. First, the available microbial larvicide formulations have a short effective period and require re-treatment of aquatic habitats every 7–10 days [22–27]. Apart from logistic concerns, repeated larvicide applications are usually associated with high material and operational costs, so the current formulations may not be affordable for large-scale use in malaria endemic areas, especially in many African countries [37].

### Development of long-lasting larvicide formulation

Slow-release briquet formulations of *Bti*/*Bs* have been developed and tested since the 1980s [12, 27]. The earlier granular formulation of *Bti*/*Bs* controlled *Aedes aegypti* in abandoned tires for 2–4 weeks, and *Bti*/*Bs* briquets exhibited larvicidal activity in large containers for 4–11 weeks. Since then, different formulations have been developed, with effective periods ranging from two weeks to six months. However, all of these tests were focused on *Aedes* larvae, and the vast majority of the tests were conducted in container environments. The few field trials conducted in urban areas yielded an effective period of 4–6 weeks, far better than the conventional 7-day formulation, but this is not sufficient for large-scale applications. Furthermore, the potential effectiveness of these long-lasting formulations of *Bti*/*Bs* against *Anopheles* larvae is unclear. The small confined environment where *Aedes* mosquitoes inhabit is very different from open field habitats where *Anopheles* mosquitoes breed.

### Prospects of long-lasting larvicide in malaria control and elimination

Recent advancements in microbial larvicide formulation, a better understanding of larval habitat productivity, and the ability to predict productive larval habitats may help strengthen the role of microbial larvicides in malaria control. First, the new formulation of EPA-approved long-lasting microbial larvicides (LLML) allow a slow release of larvicide and increase the effective period by 4–6 months and can reduce both indoor and outdoor vector density in small cluster randomized controlled trials (Box 1) [26]. Large-scale intervention (32 clusters with 16 intervention clusters) in western Kenya shows that one application of LLML could reduce 60–80% of the pupae production for ten weeks. In addition, it shows no impact on non-targeted organisms (Box 2) [38, 39]. Compared to the weekly habitat re-treatment required by conventional microbial larvicides, the 4–6-month or even 10-week re-treatment interval of LLML is a significant improvement. Although the material cost of LLML is more expensive than the conventional formulation, its long-lasting effects and infrequent re-treatment requirements may reduce the overall material and operational costs. Second, based on topographic features and satellite images, larval habitats are found to be spatially clustered [40–45]. The clustering pattern of habitats greatly facilitates the field operation of larval control through application of larvicides in targeted hotspots. New microbial larvicide formulations that can last for one to three months across different types of habitats have been field-tested [26, 38, 39]. Large-scale clustered-randomized field trial is ongoing in western Kenya [46]. Field cohort study indicated that LLML significantly reduced immature malaria vector population density and did not have detectable effect on non-targeted organisms (Box 2) [38].

Box 1. Tests of LLML efficacy and effective duration The microcosm and field tests were conducted from 2009 to 2012 to determine the impact of LLML on vector abundance [26]. The formulation the researchers tested is an EPA-approved FourStar 180-day briquettes manufactured by Central Life Sciences, Schaumburg, Illinois. The active ingredients of FourStar 180-day briquettes are *Bacillus thuringiensis israelensis* (*Bti*) strain BMP 144 (1% in weight) and *Bacillus sphaericus* (*Bs*) strain AML614 (6% in weight). This product is being marketed in the US for mosquito vector control.

**Microcosm test of effective duration**

**Fig. 1:**
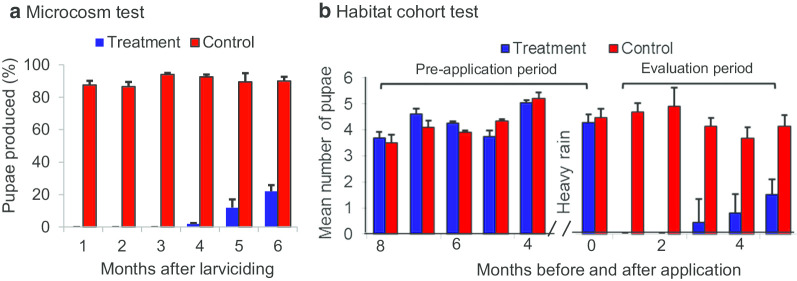
Experimental tests of long-lasting larvicide, Central Life Sciences Fourstar^®^ briquette for malaria vectors in western Kenya highland. **a** Microcosm test to determine the effective duration in *An. gambiae* larval killing; **b** Testing the effectiveness of the long-lasting larvicide using a cohort of larval habitats. Larvicide was applied in Month 0 (Original figure: Figs. 4 and 5 in Afrane et al. [26])

In this experiment, a *Bti*/*Bs* briquette was placed in a 200 L water tank filled with rainwater and the tank was covered with fine mesh. The water was tested monthly for a period of 6 months to determine the effective duration [26]. The result indicated that LLML totally inhibited mosquito pupal production in the first three months, and then reduced pupal productivity by 87.2%–98.0% for 4–6 months after application (Fig. 1a).

**Field test of efficacy and effective duration**

To test the efficacy and effective duration of the larvicide under field conditions, based on five months monitoring, 79 stable (defined as covered with water for at least 2 weeks) and productive (defined as containing larvae and pupae) habitats were selected for *Bti*/*Bs* treatment experiments, with 41 treatment and 38 control habitats in western Kenya. Results indicated that LLML reduced malaria vector pupal productivity by 100% in the first two months and then by 63.4%–90.2% for 3–5 months after application (Fig. 1b).

**Small-scale cluster-randomized trial**

**Fig. 2:**
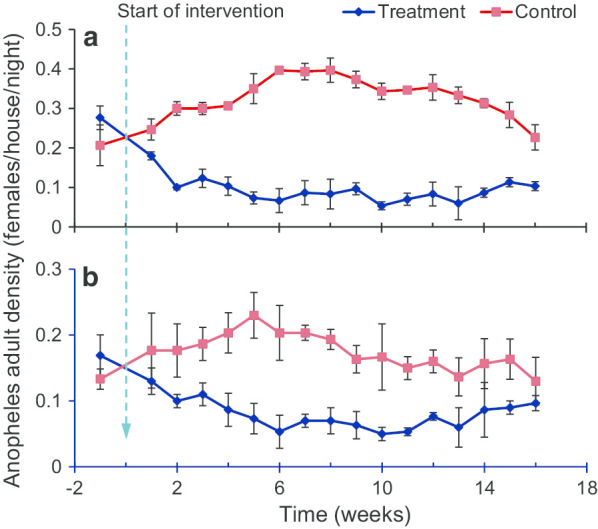
Matched cluster randomized tests of long-lasting larvicide, Central Life Sciences Fourstar^®^ briquette for malaria vectors in western Kenya highland. *Anopheles* adult densities in treatment and control groups by: **a** indoor collection, **b** outdoor collections. Larvicide was applied in week 0 (Reproduced from: Fig. 7 in Afrane et al. [26])

To test if LLML reduces indoor and outdoor *Anopheles* adult densities, a clustered-randomized six cluster trial was undertaken in three areas of western Kenya. The briquettes were applied in all larval breeding sites. Mosquito abundance indoor and outdoor was monitored weekly using the CDC miniature light traps. The application of LLML caused a 66–88% (average 80%) relative reduction in the indoor *Anopheles* density (Fig. 2a) and a 41–79% (average 65%) relative reduction in the outdoor *Anopheles* density (Fig. 2b) from week 2 to week 16. These data suggest that LLML was effective in controlling malaria vectors both indoors and outdoors in the field for several months in western Kenya.

- *Bti*: *Bacillus thuringiensis israelensis*
- *Bs*: *Bacillus sphaericus*
- CDC: Centers for Disease Control and Prevention
- LLML: Long-lasting microbial larvicide

Box 2. Impact of LLML on non-targeted organisms and vector larval populations As part of the ongoing large-scale clustered-randomized field trial conducted in western Kenya [46], field cohort study of LLML treated and control habitats were randomly selected from intervention and control clusters. The LLML was the same as described in Box 1. Larval habitat surveys were conducted weekly started 5 weeks before intervention and continued for 21 weeks after LLML intervention [38, 39]. The collected non-target organisms were classified to order and common names, malaria *Anopheles* vectors was pooled in treatment and control clusters. The study was conducted from December 2015 to December 2016.

**Impact of LLML application on non-targeted organisms**

**Fig. 3:**
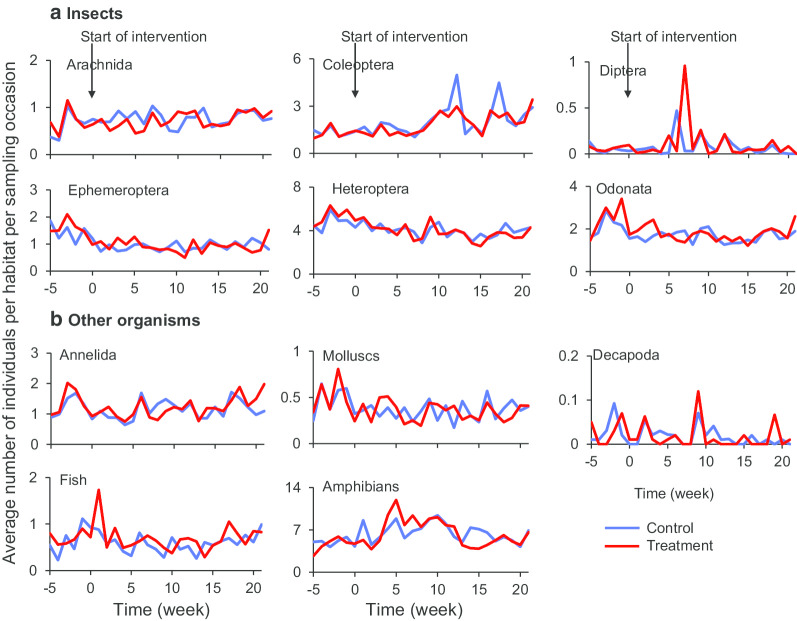
Abundance of individual taxa of non-targeted organisms in treated and control mosquito larval habitats. **a** Insects, **b** other organisms. Larvicide was applied in week 0 (Reproduced from: Fig. 3 in Derua et al. [38])

Application of LLML had no impact on the abundances of all non-targeted organisms collected during the entire study period (Fig. 3). In addition to abundance, diversity of taxa of non-target organisms was also not significantly different in the treated and control larval habitats. Likewise, taxa richness before and after application of LLML, and between treated and control larval habitats were not significantly different [38]. These results indicated that application of LLML had no impact on both abundances and species diversities of non-targeted organisms.

**Large-scale cluster-randomized trial**

**Fig. 4:**
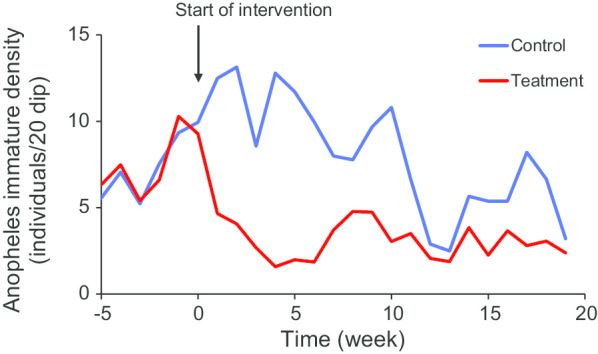
Changes in *Anopheles* immature density in treatment and control areas before and after LLML application (Reproduced from: Fig. 2 in Kahindi et al. [39])

Significant reduction in vector larval population density has been observed by week two of post-intervention (Fig. 4). There was about 70% reduction in pooled immature vector density by week four, 50% by week 12 and reduction in immature vector density was still significant by 20 weeks post-intervention (Fig. 4) [38, 39]. The reduction in old larvae (3rd–4th instar) was more pronounced than that in young larval [39].

- LLML: Long-lasting microbial larvicide.

### Issues related to field implementations

Before LLML can be implemented on a large scale, improvements must be made on both the LLML formulation and the field implementation techniques. Antonio-Nkondjio et al. has outlined some of the important guidelines for the implementation of larval control interventions [27]. Several outstanding issues with long-lasting microbial larvicides need investigations. First, what is the effective duration of the long-lasting formulation in the field? This is obviously related to the formulation, local larval ecology, habitat types, rainfall, and other ecological and environmental factors [47]. For example, fluctuating rainfall can dilute the active ingredient in larval habitats and thus may reduce the killing effect of the LLML briquettes. Second, what is the optimal application strategy and subsequent cost-effectiveness of the application strategy? Optimized application can save both cost and time. Optimal timing of applications is crucial to maximize the effectiveness of the intervention. Third, how to seamlessly integrate LLML into the national malaria intervention strategy? Some malaria endemic African countries such as Ethiopia have already incorporated larviciding into their malaria control policy [48], but how to best integrate larviciding with other intervention methods and maximize the benefit of integrated intervention remains unclear.

The cost-effectiveness of LLML should also be evaluated in other places than African countries. So far, LLML has only been evaluated in Kenya, although the conventional formulation of the same microbial larvicides has been tested/used and cost has been evaluated in other African [22–29, 35–40, 46] as well as Asian countries [49–52]. The results have been shown to be promising across different ecological settings, which is especially relevant in low transmission areas that are targeting the goal of malaria elimination [53].

Resistance is another concern for LLML. So far, *Bti*/*Bs* resistance has not been documented in laboratory or in field *Anopheles* mosquitoes although it has been tested widely for controlling malaria vectors [27, 39, 40, 54]. The decade long use of conventional *Bti*/*Bs* formulation with a short effective duration for mosquito control in the USA and Europe has not led to high resistance in mosquitoes [35, 55]. However, LLML can be effective up to 4–6 months, this chronic selection pressure may help selecting *Bti*/*Bs* resistance. On the other hand, *Bti*/*Bs* has multiple toxin components, rendering resistance difficult to evolve [12]. Nevertheless, chronic exposure and imperfect killing of LLML presents a real risk for mosquitoes to evolve resistance.

Lastly, safety is a major concern regarding field application of any insecticides regardless of chemical or biological [56]. It is generally agreed that Bti/Bs is not harmful to human and other non-targeted organisms. However, previous evaluations are mainly based on conventional formulations with a short effective duration. As mentioned earlier, LLML may pose acute chronic selection pressure on mosquitoes and other non-targeted organisms in the aquatic habitats. Recent field study showed that LLML has no impact on population abundances and biodiversity of non-targeted organisms in Kenya [38]. Additional monitoring and evaluation on the long-term effects on non-targeted organisms is needed. This is especially important in field applications.

## Conclusions and future directions

Long-lasting microbial larviciding represents a promising new tool that complements the currently front-line LLIN and IRS programs. It targets both indoor and outdoor transmission and alleviates the problem of insecticide resistance. Given the progress made in microbial larvicide formulation in conjunction with our improved understanding of mosquito ecology, in our opinion, long-lasting microbial larviciding may be a cost-effective supplemental malaria control method.

Before we can scale-up the LLML program, we need better understanding on some outstanding questions as described below:

1. Are LLML suitable for different ecological settings? In other words, how does environmental factor impact the effectiveness of LLML? The effectiveness of LLML may depend on local larval ecology, i.e., is it possible that LLML is more effective in some habitat types than the others?
2. Conventional formulation of microbial larvicide has been shown effective in some settings, but it is costly. Is LLML cost-effectiveness for scale-ups?
3. So far, no Bti/Bs resistance in Anopheles mosquitoes has been reported after decades of application, potentially due to its multiple toxin components. However, with the persistent and chronic selection pressure from LLML exposure, will mosquitoes develop rapid resistance to Bti/Bs?
4. It is generally believe that Bti/Bs has no toxicity to people, various tests revealed no detectable harm to non-target organisms. Will persistent exposure to LLML cause harm to other organisms in the aquatic habitats?

## Data Availability

All data and results are already appeared in the paper.
